# Channel-Focused Modified TURP for Post-Radiation Bladder Outlet Obstruction: A Case Series

**DOI:** 10.3390/jcm15208168

**Published:** 2026-10-20

**Authors:** Jeong Hyun Lee, Jang Hwan Kim, Kyung Tak Oh

**Affiliations:** Department of Urology, Severance Hospital, College of Medicine, Yonsei University, Seoul 03722, Republic of Korea; spiralcloud@yuhs.ac (J.H.L.);

**Keywords:** post-radiation bladder outlet obstruction, modified transurethral resection of the prostate, prostate radiotherapy complications, lower urinary tract symptoms, urinary incontinence outcomes

## Abstract

**Background/Objectives:** Bladder outlet obstruction (BOO) after prostate radiotherapy is surgically challenging because of radiation-induced tissue changes and functional complications. We evaluated the efficacy and safety of channel-focused modified transurethral resection of the prostate (RadTURP) for post-radiation BOO. **Methods:** This retrospective case series included 11 patients who underwent RadTURP for post-radiation BOO between 2014 and 2025. Outcomes included symptom scores, voiding parameters, and postoperative complications. Pre–post changes were assessed using the Wilcoxon signed-rank test. **Results:** Median age was 75 years and median follow-up was 29 months. The median resection proportion was 24%. IPSS improved from 27 to 10 (*p* = 0.003), QoL from 5 to 3 (*p* = 0.007), OABSS from 12 to 6 (*p* = 0.016), Qmax from 6 to 17 mL/s (*p* = 0.006), and PVR from 59 to 0 mL (*p* = 0.047). De novo urinary incontinence occurred in four patients (36.4%), including two (18.2%) with persistent stress urinary incontinence requiring artificial urinary sphincter implantation. Among six patients undergoing symptom-driven postoperative cystoscopy, five (83.3%) had stone/calcification-related findings requiring energy-free endoscopic removal. One patient (9.1%) developed a urethral stricture requiring repeated dilation. **Conclusions:** In this small case series, channel-focused RadTURP was associated with improved urinary symptoms and voiding function after prostate radiotherapy. However, de novo urinary incontinence occurred in 36.4% of patients, highlighting an important postoperative safety concern for patient counseling and selection. Larger studies are needed to define durability and safety.

## 1. Introduction

Radiation-based therapies, including external beam radiotherapy (EBRT) and brachytherapy, are widely used to treat localized prostate cancer. With longer post-treatment follow-ups and an aging patient population, an increasing number of patients present with lower urinary tract symptoms (LUTS) and bladder outlet obstruction (BOO) after radiation therapy [1,2,3]. In this setting, transurethral resection of the prostate (TURP) remains one of the most commonly reported surgical options for prostate-related BOO, although the optimal approach in previously irradiated patients remains uncertain [4,5].

Histopathologic studies of prostate biopsy specimens after radiotherapy have described epithelial injury, stromal remodeling, vascular changes, glandular atrophy, and increased fibrosis [6,7,8]. These biopsy-based histologic findings should be distinguished from the gross endoscopic tissue characteristics encountered intraoperatively during TURP, which represent a different type of observation. Consequently, surgeons must balance adequate channel creation and hemostasis against the preservation of continence mechanisms because aggressive resection may increase sphincteric/urethral injury, whereas overly conservative resection may result in insufficient relief or early recurrence [9]. Prior studies have reported variable outcomes after post-radiation TURP, with urinary incontinence and urethral complications remaining notable risks in certain subsets [1,2,3,9]. More recent literature has similarly emphasized the limited and heterogeneous evidence regarding desobstructive surgery after prostate radiotherapy, with postoperative urinary incontinence remaining an important concern [4,5,10]. Although alternative transurethral approaches such as photoselective vaporization and HoLEP have been described, comparative evidence remains insufficient to establish an optimal surgical strategy for previously irradiated patients [4,5,10].

Despite these concerns and the growing literature on post-radiation outlet surgery, procedural decision-making remains poorly standardized, particularly regarding the extent and manner of resection in irradiated prostatic tissue. Importantly, TURP performed in the post-radiation setting may need to differ from conventional TURP for benign prostatic hyperplasia, given the distinct tissue characteristics and heightened risks of functional complications. Accordingly, we adopted a modified TURP technique tailored to post-radiation anatomy (RadTURP) and tissue properties, and sought to evaluate its efficacy and safety. As detailed descriptions of intraoperative tissue features and their relationship with postoperative function and complications remain limited, this study assessed the operative findings, functional outcomes, and complications among patients undergoing RadTURP after prostate radiation therapy, including both EBRT and brachytherapy. By clarifying the intraoperative patterns and outcome profiles in this unique clinical context, we aimed to develop safer and more consistent surgical strategies and improve patient counseling.

## 2. Materials and Methods

### 2.1. Study Design and Patient Selection

This study was approved by the Institutional Review Board of our institution (approval no. 4-2026-0189; approved on 7 April 2026), and the requirement for informed consent was waived due to the retrospective study design. This retrospective case series was conducted at a single tertiary referral center. We reviewed the medical records of patients who underwent RadTURP for BOO following prostate radiation therapy between 1 January 2014, and 30 September 2025. Eligible patients had previously received definitive radiation therapy for prostate cancer, including EBRT or brachytherapy, and had subsequently developed worsening LUTS suggestive of BOO. The diagnosis of BOO was confirmed using pressure flow studies and cystoscopic evaluation. Cystoscopy was performed at least 1 year after the completion of radiation therapy to minimize the influence of early post-radiation inflammatory changes. Patients were excluded if the postoperative follow-up was shorter than 3 months or if essential clinical data were incomplete. Baseline demographic and clinical variables, including age, comorbidity status, radiation modality, interval between radiation therapy and RadTURP, prostate volume, and preoperative urinary function parameters, were collected from electronic medical records. For calculation of the resection proportion, prostate volume documented at the time of radiation therapy was used because contemporaneous prostate volume measurements immediately before RadTURP were unavailable. Radiotherapy-related variables included treatment modality, available dose and fractionation data, treatment technique, isotope and implantation details for brachytherapy, and the interval between radiation therapy and RadTURP. When radiotherapy was performed at an outside institution, treatment details were recorded as unavailable if the original radiotherapy records could not be obtained. Prior local treatment for prostate cancer in addition to radiotherapy was not an exclusion criterion. One patient (9.1%) had undergone HIFU before subsequent radiotherapy.

### 2.2. Surgical Technique

The procedures were performed by two urologists with 4 to more than 20 years of experience performing TURP. Both surgeons followed the same channel-focused resection strategy. All procedures were performed using a 24Fr transurethral resection system (OES Pro Resectoscope; Olympus Corp., Tokyo, Japan). A narrow sheath was preferentially used to minimize the mechanical trauma to the urethral sphincter. In contrast to conventional TURP performed for benign prostatic hyperplasia, the surgical goal in RadTURP was not complete adenoma removal but rather restoration of urethral patency. Resection was selectively performed at the bladder outlet and prostatic urethra to remove the most obstructed tissue. Because irradiated prostate tissue often demonstrates dense fibrosis and reduced compressibility, resection was performed in a targeted and controlled manner. A channel-oriented resection strategy was adopted to create a tunnel-like cavity through the obstructed prostatic urethra, while avoiding excessive resection near the sphincter. The macroscopic appearance of the prostate tissue was documented in the operative records. After completion of the resection, the removed tissue was manually compressed to remove the irrigation fluid and weighed to determine the resection volume. At the conclusion of the procedure, an 18Fr or 20Fr three-way Foley catheter was inserted and continuous bladder irrigation with normal saline was initiated.

### 2.3. Postoperative Management and Follow-Up

The Foley catheter was removed on postoperative day 2. Patients were evaluated for spontaneous voiding after catheter removal. If spontaneous voiding was inadequate, the patients were instructed to perform clean intermittent catheterization. Patients were followed up at 1, 3, 6, and 12 months postoperatively. At each visit, symptom and functional assessments were performed, including the International Prostate Symptom Score (IPSS), IPSS-quality of life (QoL), Overactive Bladder Symptom Score (OABSS), uroflowmetry, and postvoid residual urine volume (PVR). Postoperative cystoscopy was not routinely performed for surveillance but was selectively performed when clinically indicated by urinary symptoms or suspected postoperative complications. Postoperative complications were recorded throughout the follow-up period and classified according to the Clavien–Dindo classification system.

### 2.4. Outcome Measures

The primary outcomes were changes in urinary symptoms and voiding function after RadTURP. Symptom outcomes were assessed using IPSS, IPSS-QoL, and OABSS. The objective functional outcomes included the maximum urinary flow rate (Qmax) and PVR. The operative outcomes included operative time, resection volume, and resection proportion relative to the historical prostate volume documented at the time of radiation therapy. The resection proportion was calculated as the resected tissue weight (g) divided by the prostate volume (mL) documented at the time of radiation therapy and multiplied by 100. Because contemporaneous prostate volume measurements immediately before RadTURP were unavailable, this value was considered an approximate measure of resection extent relative to the historical prostate volume rather than the actual proportion of prostate tissue resected at the time of RadTURP. For the paired preoperative–postoperative analysis presented in Table 1, the most recent available postoperative measurement for each patient was used; therefore, the timing of the postoperative assessment varied according to the duration of follow-up. Complete paired data were available for all 11 patients for each functional outcome.

### 2.5. Statistical Analysis

Continuous variables are reported as medians with interquartile ranges (IQRs), and categorical variables as frequencies and percentages. Preoperative and postoperative functional outcomes were compared using the Wilcoxon signed-rank test. Postoperative complications and continence outcomes were descriptively summarized according to prior radiation modality (brachytherapy vs. EBRT). Given the very small subgroup sizes (brachytherapy, *n* = 8; EBRT, *n* = 3), no formal statistical comparisons between radiation modalities were performed. Statistical significance was defined as a two-sided *p*-value of <0.05. All statistical analyses were performed using IBM SPSS Statistics for Windows, version 29.0 (IBM Corp., Armonk, NY, USA). Given the exploratory nature and small sample size of this case series, no adjustment for multiple comparisons was performed. Accordingly, the reported *p*-values should be interpreted as exploratory rather than confirmatory.

### 2.6. Use of Generative Artificial Intelligence

ChatGPT (GPT-5.2; OpenAI, San Francisco, CA, USA) was used solely for English-language editing and to improve the clarity and readability of the manuscript. The authors reviewed and edited all AI-assisted content and take full responsibility for the final manuscript. Generative artificial intelligence was not used for study design, data collection, statistical analysis, or interpretation of the results.

## 3. Result

### 3.1. Patient Characteristics

The baseline demographic and clinicopathological characteristics at the time of RadTURP are summarized in Table 2. The median age was 75 (IQR, 70.5–77) years, and the median follow-up duration was 29 (IQR, 17–37.5) months. Among the 11 patients, prior prostate radiation therapy consisted of EBRT in three (27.3%) and brachytherapy in eight (72.7%). All eight patients in the brachytherapy group underwent iodine-125 permanent seed implantation. The median numbers of implantation needles and seeds were 21 (IQR, 19.5–22.5) and 89.5 (IQR, 83.3–98.5), respectively. One patient (9.1%), who was included in the EBRT group, had undergone HIFU before radiotherapy. Regarding ADT exposure, all eight patients in the brachytherapy group received short-term neoadjuvant ADT for approximately 2–3 months before brachytherapy and were no longer receiving ADT at the time of RadTURP. Among the three patients in the EBRT group, two were receiving ongoing ADT at the time of RadTURP, whereas one had not received ADT. Among the three patients treated with EBRT, detailed treatment records were available for one patient, who received intensity-modulated radiation therapy (IMRT) at a total dose of 60 Gy in 20 fractions. The remaining two patients underwent EBRT at outside institutions, and detailed information regarding radiation technique, dose, and fractionation was unavailable. Additional radiotherapy-related characteristics are presented in Supplementary Table S2.

### 3.2. Intraoperative Finding

**Figure 1:**
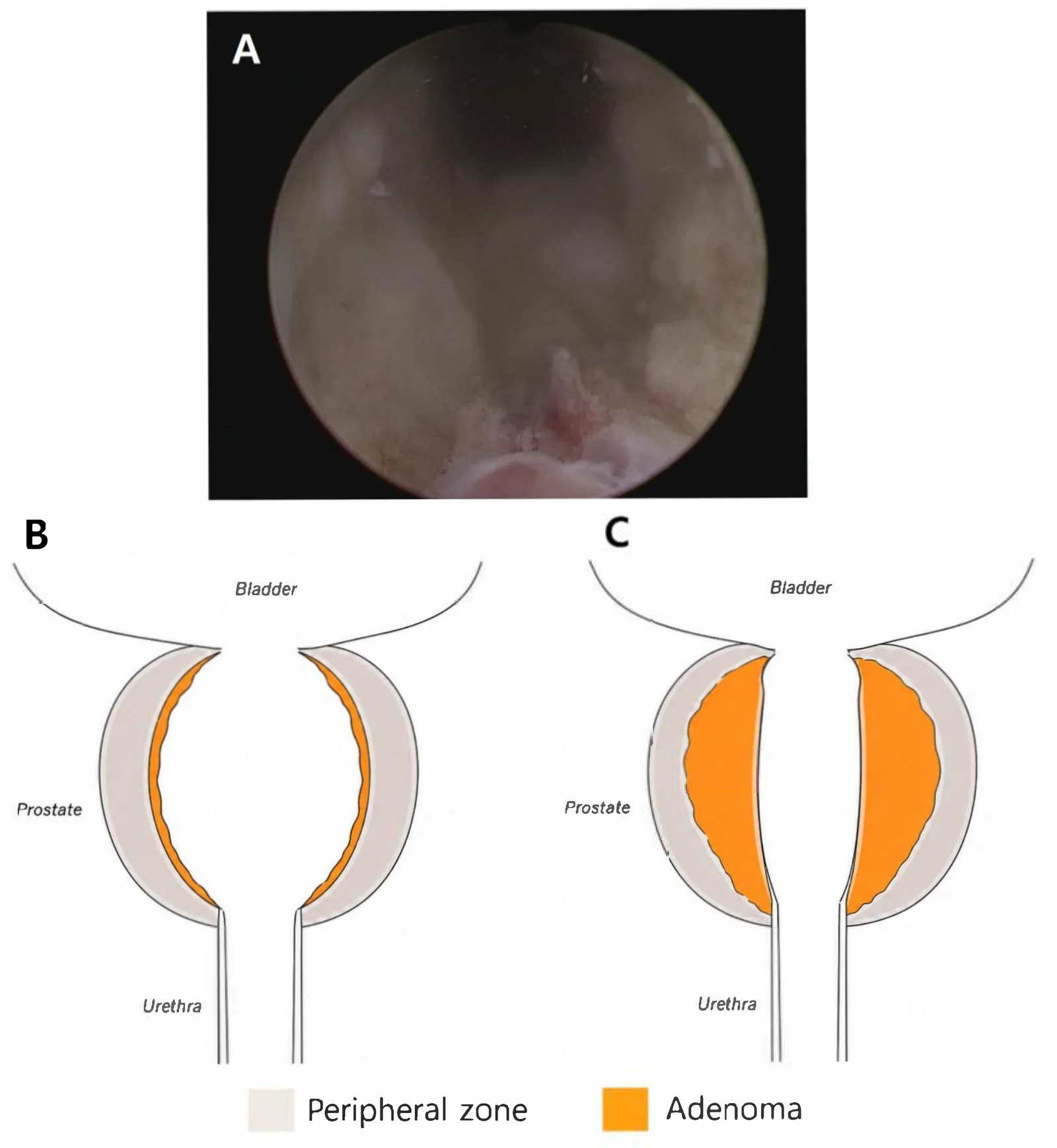
Surgical concept of RadTURP compared with conventional TURP. (**A**) Postoperative image showing the creation of a tunnel-like passage. (**B**) Schematic of conventional TURP or HoLEP. (**C**) Schematic illustration of RadTURP. Panels (**B**,**C**) are idealized illustrations intended to demonstrate the surgical concept and are not based on patient-specific imaging or histopathologic mapping. The labels “adenoma” and “peripheral zone” are used for illustrative purposes and do not represent patient-specific anatomical boundaries. Abbreviations: TURP, transurethral resection of the prostate; RadTURP, modified TURP after radiation therapy; HoLEP, Holmium Laser enucleation of the prostate.

Intraoperatively, the irradiated prostatic tissue appeared pale, dense, and fibrotic, with reduced compressibility compared to typical BPH tissue. Therefore, resection was performed in a targeted manner, focusing on the most obstructive tissue at the bladder outlet and prostatic urethra to establish a continuous channel. After selective resection, a patent tunnel-like cavity was consistently observed, as shown in the representative endoscopic images (Figure 1).

### 3.3. Perioperative Outcomes

The operative and perioperative outcomes are summarized in Table 2. The median operative time was 40 (IQR 24.5–45.5) minutes, and the median resection volume was 11 (IQR 9.5–12) g. The median resection proportion relative to the historical prostate volume documented at the time of radiotherapy was 24% (IQR 19–34%). One patient (9.1%) underwent concomitant bladder neck incision for narrowing of the bladder neck noted intraoperatively. The median follow-up duration was 29 (IQR 17–37.5) months.

### 3.4. Functional Outcomes

**Figure 2:**
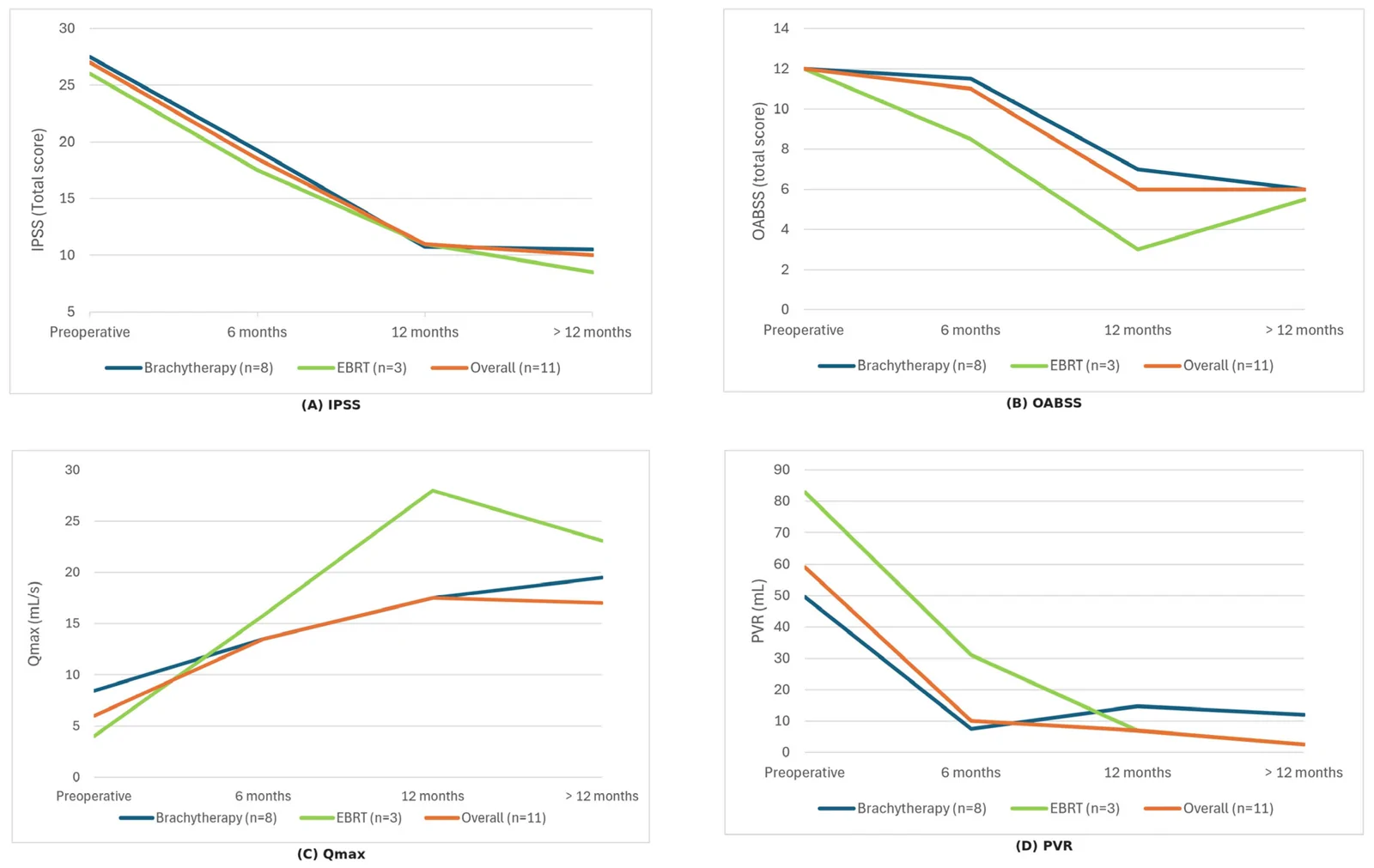
Longitudinal changes in functional outcomes after RadTURP in patients with prior radiation therapy. (**A**) IPSS, (**B**) OABSS, (**C**) Qmax, and (**D**) PVR are shown at the preoperative assessment, 6 and 12 months, and at the most recent follow-up beyond 12 months (>12 months). Values are presented as medians at each time point, calculated using available cases. The number of patients contributing to the data may differ across time points because of missing data. Of the 11 patients, 2 had follow-up limited to within 6 months, 1 was followed for 6–12 months, and 8 were followed beyond 12 months; among the latter, 5 had complete assessments at all preceding time points, whereas 3 had intermittently missing data. Modality-stratified trends were descriptive given the small EBRT subgroup (*n* = 3). RadTURP, modified transurethral resection of the prostate after radiation therapy; IPSS, International Prostate Symptom Score; OABSS, Overactive Bladder Symptom Score; Qmax, maximum flow rate; PVR, postvoid residual; EBRT, external beam radiation therapy.

Changes in functional outcomes before and after RadTURP are presented in Table 1, and longitudinal trends over the follow-up period are shown in Figure 2. Of the 11 patients, two had follow-up limited to within 6 months, one was followed for 6–12 months, and eight were followed for more than 12 months. Among the eight patients followed beyond 12 months, five had complete functional assessments at the preceding postoperative time points, whereas three had intermittently missing assessments. Accordingly, the longitudinal results in Figure 2 are based on the available observations at each time point. Symptom scores improved significantly, with a reduction in IPSS from 27 (IQR 23–29) to 10 (IQR 8–13.5) (Δ −17, IQR −21 to −7; *p* = 0.003), IPSS QoL score from 5 (IQR 4–5) to 3 (IQR 3–4) (Δ −1, IQR −2 to −1; *p* = 0.007), and OABSS from 12 (IQR 10.5–12.5) to 6 (IQR 4–9.5) (Δ −4, IQR −8 to −2; *p* = 0.016). Objective voiding parameters also improved: Qmax increased from 6 (IQR 4.75–8.95) to 17 mL/s (IQR 13.6–27.5) (Δ +11, IQR 8.1–21.2; *p* = 0.006), while PVR decreased from 59 (IQR 25.5–161.5) to 0 mL (IQR 0–30) (Δ −30, IQR −80 to −3; *p* = 0.047). Consistent with these paired comparisons at the last available postoperative assessment, Figure 2 shows that improvements in symptom scores and voiding parameters were generally observed at 6 months and were maintained for 12 months and beyond.

### 3.5. Complications

Postoperative complications are summarized in Table 3. Overall, de novo urinary incontinence occurred in four of the 11 (36.4%) patients. Of these, two (18.2%) patients developed de novo urgency urinary incontinence that was transient and resolved during follow-up, whereas two (18.2%) developed de novo stress urinary incontinence (SUI) that persisted and ultimately required artificial urinary sphincter (AUS) implantation. When stratified by radiotherapy modality, de novo incontinence occurred in two of eight patients (25.0%) after brachytherapy and two of three patients (66.7%) after EBRT; persistent SUI requiring AUS occurred in one (12.5%) patient in the brachytherapy group and one (33.3%) in the EBRT group.

Gross hematuria occurred in four patients (36.4%), all of whom had undergone brachytherapy. Three patients (27.3%) reported mild, transient episodes of gross hematuria during outpatient follow-up that resolved without intervention; event-specific hemoglobin measurements were not available for these episodes, and none required blood transfusion. One patient (9.1%) developed hematoma with acute urinary retention 2 months after RadTURP and underwent cystoscopic clot evacuation using an Elik evacuator. In this patient, hemoglobin decreased from 10.7 to 7.8 g/dL; however, no active bleeding was identified during cystoscopy, and neither endoscopic cauterization nor blood transfusion was required.

Six of the eleven patients (54.5%) underwent postoperative cystoscopy. Among these six patients, stone/calcification-related findings, including stones, calcified lesions, or stone-like debris, were identified in five (83.3%), corresponding to five of the overall eleven patients (45.5%). All five patients underwent energy-free endoscopic removal. On subsequent follow-up cystoscopy, the previously identified stone/calcification-related findings had resolved, with no residual stone or calcified material observed.

One patient (9.1%) developed a clinically significant urethral stricture requiring intervention. This patient had previously undergone HIFU and EBRT and developed postoperative voiding difficulty with failure of flexible cystoscopic passage. Urethral dilation up to 22 Fr was performed on three occasions. This was the same patient who subsequently developed persistent urinary incontinence requiring AUS implantation. No patient developed recurrent urinary retention requiring catheterization.

## 4. Discussion

In this study, RadTURP performed after prior radiation therapy was associated with significant improvements in voiding-related outcomes. Endoscopically, irradiated prostates commonly demonstrate dense, fibrotic tissue characteristics distinct from those of typical BPH; this observation informed our operative strategy. Rather than pursuing extensive adenoma removal, we performed a modified channelization-focused TURP aimed at restoring urethral patency through selective removal of the most obstructive tissue, as illustrated by the representative cystoscopic findings and the postoperative tunnel-like cavity (Figure 1). Following this approach, the symptom burden decreased substantially, with significant reductions in IPSS, IPSS-QoL, and OABSS scores, while the objective voiding parameters improved, including an increase in Qmax and a decrease in PVR. Together, these findings suggest that this channel-focused RadTURP approach may provide clinically meaningful relief from radiation-associated BOO and LUTS while limiting unnecessary deep or wide resection in tissues vulnerable to poor healing and sphincter injury.

Regarding safety, postoperative urinary incontinence was a clinically important adverse outcome in this small case series. De novo urinary incontinence occurred in four of the 11 patients (36.4%): two (18.2%) experienced transient urgency-predominant leakage that resolved during follow-up, whereas two (18.2%) developed persistent stress-predominant incontinence requiring AUS implantation. Notably, one of the two patients who required AUS implantation had previously undergone HIFU, TURP, and radiation therapy, representing a history of multiple local prostate-directed treatments and prior outlet surgery that may have increased the baseline risk of sphincteric dysfunction. Nevertheless, the relative contributions of these prior treatments and the subsequent RadTURP to postoperative incontinence cannot be determined in this small case series. Thus, despite the observed improvements in urinary symptoms and voiding parameters, the relatively high rate of de novo incontinence, including persistent SUI requiring AUS implantation in two patients, represents an important safety consideration when counseling patients regarding outlet surgery after prostate radiotherapy.

The relatively high rate of de novo incontinence in this setting likely reflects not only the effect of the outlet surgery itself but also the underlying impact of radiation on the continence mechanism. Pelvic radiotherapy may induce structural changes in the urethra and periurethral tissues and compromise the sphincteric reserve through fibrosis, reduced vascularity, and impaired tissue compliance and healing even before outlet surgery [11]. In some patients, BOO may partially mask this latent sphincter insufficiency. Once the obstruction is relieved, stress-predominant leakage may become clinically apparent. This mechanism may explain why postoperative SUI can occur even after deliberately limited, channel-focused resection and underscores the importance of careful preoperative counseling regarding continence-related risk in patients undergoing outlet surgery after prostate radiotherapy.

Another notable finding was the frequent occurrence of postoperative stone/calcification-related lesions among patients who underwent symptom-driven cystoscopic evaluation. Five of the six patients who underwent postoperative cystoscopy had stones, calcified lesions, or stone-like debris. In our practice, these lesions were managed by energy-free endoscopic removal, deliberately avoiding additional electrocautery or resection of the irradiated tissue. Subsequent follow-up cystoscopy, when performed, showed no residual stone/calcification-related lesions. This approach may be particularly relevant in previously irradiated tissue, in which additional thermal injury or extensive resection could potentially perpetuate tissue necrosis and delayed healing. Although evidence regarding the optimal management of postoperative dystrophic calcification is limited, previous case reports after TURP have similarly described recurrent calcification after resection or laser treatment and subsequent improvement after cautious removal without electrical energy to minimize further tissue trauma [12]. Thus, in addition to limiting the extent of the initial resection, minimizing additional tissue injury during the management of postoperative calcification may represent another component of a tissue-preserving strategy in irradiated prostates. However, given the small number of selectively cystoscoped patients in our series, this observation should be considered hypothesis-generating rather than evidence of the superiority of energy-free removal.

Management of LUTS and BOO after prostate radiotherapy should be individualized according to symptom severity, the predominant storage or voiding component, bladder emptying, and the anatomical level of obstruction. Post-radiation LUTS may reflect a combination of bladder dysfunction, outlet obstruction, urethral pathology, and sphincteric dysfunction, and the available evidence specifically addressing treatment in this population remains limited [13]. ADT can reduce prostate volume and may improve LUTS or BOO attributable to benign prostatic enlargement. In our cohort, all eight patients treated with brachytherapy had received short-term neoadjuvant ADT before brachytherapy but were no longer receiving ADT at the time of RadTURP, whereas two of the three patients treated with EBRT remained on ADT at the time of RadTURP. Nevertheless, post-radiation BOO is likely multifactorial and may involve radiation-induced fibrosis, reduced tissue compliance, and urethral or bladder neck pathology in addition to prostate enlargement. Therefore, the development or persistence of BOO despite prior or ongoing ADT may reflect structural components of obstruction that are not expected to be reversed by androgen suppression, rather than simply indicating a lack of response to ADT. Accordingly, conservative and pharmacologic management may be considered in patients without a clear indication for surgical intervention, whereas temporary catheterization or clean intermittent catheterization may be required in patients with urinary retention or inadequate bladder emptying. When an anatomical urethral or bladder neck stenosis is identified, treatment should be directed toward the specific site of obstruction. For persistent prostatic outlet obstruction refractory to conservative management, reported transurethral options include TURP, photoselective vaporization, and, in selected patients, HoLEP [4,10]. Notably, Titus et al. recently reported the feasibility of en-bloc HoLEP in patients with a history of prostate radiation therapy or HIFU, further illustrating the potential role of laser enucleation in carefully selected patients [10]. However, evidence directly comparing these procedures in previously irradiated patients remains limited, and no single surgical approach has been established as optimal [4].

Outlet surgery after radiotherapy is particularly challenging because radiation-induced fibrosis, vascular injury, reduced tissue compliance, and impaired healing may increase the risks of sphincteric dysfunction, recurrent stenosis, bleeding, tissue necrosis, and other postoperative morbidity [14]. Consistent with these concerns, one patient in our cohort developed a clinically significant postoperative urethral stricture requiring repeated dilation. A recent scoping review specifically examining bladder outlet obstruction surgery in the setting of prostate radiotherapy found that the available evidence remains limited and heterogeneous, with urinary incontinence representing an important adverse outcome after post-radiation desobstructive surgery [5]. Fistula formation is an uncommon but serious complication in previously treated or surgically manipulated tissues, and rare cases have also been reported after outlet procedures such as HoLEP [14,15]. These considerations highlight the importance of identifying the underlying cause of LUTS and BOO, individualized selection of the outlet procedure, and careful preoperative counseling regarding functional morbidity. In this context, our RadTURP strategy was designed to restore urethral patency while limiting unnecessary tissue removal and sphincter-adjacent manipulation rather than attempting complete adenoma resection.

A key strength of the present study is that it provided real-world data on the management of BOO after contemporary prostate radiotherapy. The use of diverse radiation modalities continues to expand, including the growing clinical adoption of carbon-ion/heavy-ion radiotherapy with the establishment of new facilities. Post-radiation BOO and its surgical management are increasingly relevant in survivorship care [16]. Notably, however, our cohort predominantly consisted of patients previously treated with brachytherapy (8/11, 72.7%), whereas only three patients had undergone EBRT. Therefore, the findings of this small case series may be more representative of post-brachytherapy BOO than of post-radiation BOO across all radiotherapy modalities. Current EAU guidelines recommend good baseline urinary function, including an IPSS < 12 and Qmax > 15 mL/s, when considering LDR brachytherapy monotherapy. However, IPSS and uroflowmetry were not routinely obtained as part of the pre-brachytherapy evaluation in our cohort, and complete pre-brachytherapy IPSS and Qmax data were therefore unavailable. The available measurements had been obtained selectively during urologic evaluation for pre-existing BPH rather than as standardized assessments for brachytherapy eligibility. Consequently, these incomplete data cannot reliably determine whether the brachytherapy-treated patients in our cohort met these urinary function criteria before treatment.

In addition, our study adds a clinically relevant risk–benefit perspective by jointly quantifying functional gains and postoperative adverse events, whereas prior post-brachytherapy TURP series have largely emphasized continence outcomes and retreatment risk. In the present case series, de novo urinary incontinence occurred in 4 of 11 patients (36.4%). Previous post-brachytherapy TURP series reported urinary incontinence rates of 25.3% in Mock et al. and 18% in Kollmeier et al. [1,17]. Mock et al. further reported that incontinence occurred in 19% of patients after a single TURP and increased to 53% among those undergoing multiple resections, while Kollmeier et al. observed a higher incontinence rate among patients undergoing TURP more than 2 years after implantation (36% vs. 8%) [1,17]. In our cohort, two patients (18.2%) developed persistent SUI requiring AUS implantation, underscoring that continence-related morbidity can be substantial even with a deliberately limited resection strategy. Among the eight patients previously treated with brachytherapy, de novo urinary incontinence occurred in two patients (25.0%). Given the very small subgroup sizes, this observation should be considered descriptive and should not be interpreted as evidence of differences in incontinence risk according to radiation modality or relative to previous series. Direct comparison of incontinence rates across studies is also limited by differences in patient selection, radiation modality, definitions of urinary incontinence, number of outlet procedures, and duration of follow-up. Importantly, beyond continence outcomes, we quantified **improvements** in both patient-reported and objective voiding measures using paired analyses (IPSS, IPSS-QoL, OABSS, Qmax, and PVR), thereby providing additional information for counseling and shared decision-making in this high-risk population.

A practical contribution of this study is an explicit description of a tissue-tailored resection concept for irradiated prostate tissues. Unlike conventional TURP for BPH, which often requires broader adenoma removal, RadTURP is deliberately limited to the creation of a patent channel through an obstructed prostatic urethra. This distinction may be particularly important in irradiated tissues where dense fibrosis and reduced compliance can increase the risk of unnecessary sphincter-adjacent injuries during aggressive resection.

The median resection proportion relative to the historical prostate volume documented at the time of radiotherapy was 24% (IQR 19–34%). Because contemporaneous prostate volume measurements immediately before RadTURP were unavailable and prostate volume may change following radiotherapy, this value should be regarded as an approximate indicator of resection extent rather than the actual proportion of prostate tissue removed at the time of RadTURP. Greater resection proportions have been reported in conventional TURP series for BPH; [18] however, direct quantitative comparison with our cohort is not appropriate because of differences in patient population, surgical context, and, importantly, the timing and methods of prostate volume assessment. Nevertheless, the relatively small amount of resected tissue, together with the operative strategy described above, is consistent with our intended approach of targeted channel creation rather than extensive adenoma removal. Improvements were observed across both symptom-based and objective measures (IPSS, IPSS-QoL, OABSS, Qmax, and PVR). By pairing these functional outcomes with a detailed description of the operative concept and representative endoscopic findings (Figure 1), our study provides practical information regarding a tissue-tailored approach to outlet surgery in irradiated prostates.

This study had some limitations. First, this was a retrospective, single-center case series, and decisions regarding surgical indication, operative judgment, and follow-up intensity may reflect local practice patterns; therefore, selection bias cannot be excluded. This is particularly relevant after radiotherapy, where post-treatment lower urinary tract dysfunction and BOO represent a heterogeneous clinical spectrum and where the existing literature is frequently limited by variability in study designs, endpoints, and adverse event definitions, complicating cross-study interpretation. Second, the small sample size (*n* = 11) and limited follow-up reduced the statistical power and may have limited our ability to capture clinically important late events, including persistent incontinence, urethral/outlet stenosis or recurrent obstruction, and delayed bleeding outcomes that can emerge over longer time horizons in irradiated tissue. In addition, multiple functional outcomes were evaluated without adjustment for multiple comparisons, which increases the risk of type I error. Therefore, the observed *p*-values should be interpreted cautiously and considered exploratory rather than confirmatory. Because the EBRT subgroup was very small, any modality-based comparison is necessarily exploratory and should be interpreted with caution. Furthermore, postoperative cystoscopy was not routinely performed for surveillance but was selectively performed in symptomatic or clinically indicated patients. Consequently, stone/calcification-related findings and urethral complications may have been underdetected in patients who did not undergo postoperative cystoscopy, while their observed frequency among cystoscopically evaluated patients may have been influenced by indication bias. In addition, contemporaneous prostate volume measurements immediately before RadTURP were unavailable, and the calculated resection proportion was based on the historical prostate volume documented at the time of radiotherapy. Given potential changes in prostate volume after radiotherapy, this value may not accurately represent the actual proportion of prostate tissue resected at RadTURP. Third, although the inclusion of both EBRT and brachytherapy reflects real-world practice, heterogeneity in radiotherapy doses/techniques may influence tissue injury patterns and complication risks. Radiation-associated effects such as fibrosis, vascular injury, chronic inflammation, and impaired healing can contribute to complex outlet pathology and may affect postoperative toxicity and functional recovery [19]. Accordingly, future studies should incorporate standardized radiotherapy parameters to enable a more refined risk stratification.

In conclusion, this small case series showed that the RadTURP strategy was associated with significant improvements in urinary symptoms and voiding function in patients with BOO after prostate radiation therapy. Intraoperatively, the irradiated prostatic tissue consistently demonstrated dense, fibrotic, and poorly compressible features, supporting a tissue-tailored, channel-focused approach rather than extensive adenoma resection. However, de novo urinary incontinence occurred in 36.4% of patients, and two patients (18.2%) developed persistent SUI requiring AUS implantation, highlighting the clinically important continence-related morbidity associated with outlet surgery in this setting. Therefore, the potential functional benefits of RadTURP should be balanced against the risk of postoperative incontinence, with careful patient selection and preoperative counseling. Larger studies with longer follow-up are needed to better define the durability, safety, and appropriate patient selection for this approach.

## Figures and Tables

**Table 1:** Changes in functional outcomes before and after RadTURP in patients with prior radiation therapy.

	Preoperative	Postoperative	Δ (Postoperative–Preoperative)	*p*-Value
IPSS	27 (23–29)	10 (8–13,5)	−17 (−21–−7)	0.003
QoL	5 (4–5)	3 (3–4)	−1 (−2–−1)	0.007
OABSS	12 (10.5–12.5)	6 (4–9.5)	−4 (−8–−2)	0.016
Qmax	6 (4.75–8.95)	17 (13.6–27.5)	11 (8.1–21.2)	0.006
PVR	59 (25.5–161.5)	0 (0–30)	−30 (−80–−3)	0.047

Complete paired preoperative and most recent postoperative data were available for all 11 patients for each outcome shown; therefore, all 11 patients were included in each paired comparison. RadTURP, modified transurethral resection of the prostate after radiation therapy; IPSS, International Prostate Symptom Score; QoL, quality of life; OABSS, Overactive Bladder Symptom Score; Qmax, maximum urinary flow rate; PVR, postvoid residual.

**Table 2:** Baseline characteristics and operative variables of patients undergoing RadTURP after prostate radiation therapy.

Total Number	11
Age at RadTURP, years	75 (70.5–77)
Charlson comorbidity index, score	6 (5–6.5)
Follow-up duration, months (median [IQR])	29 (17–37.5)
Radiation modality, *n* (%)	
Brachytherapy	8 (72.72%)
EBRT	3 (27.27%)
ADT exposure, *n* (%)	
Prior ADT	10 (90.91%)
Ongoing ADT at RadTURP	2 (18.18%)
Interval from radiation therapy to RadTURP, months	13 (12.5–20.5)
Baseline prostate data	
Prostate volume at the time of radiotherapy, mL	49 (36.5–55)
Operative variables	
Operative time, minutes	40 (24.5–45.5)
Resection volume, g (or mL)	11 (9.5–12)
Resection proportion relative to historical prostate volume, %	24 (19–34)
Additional intraoperative procedures, *n* (%)	1 (9.1%)

Data are number (%) and median (interquartile range). RadTURP, modified transurethral resection of the prostate after radiation therapy; IQR, interquartile range; EBRT, external beam radiation therapy.

**Table 3:** Postoperative complications.

	Overall (*n* = 11)	Brachytherapy (*n* = 8)	EBRT (*n* = 3)
De novo urinary incontinence	4 (36.36%)	2 (25%)	2 (66.67%)
Transient (resolved)	2 (18.18%)	1 (12.5%)	1 (33.33%)
Persistent requiring AUS	2 (18.18%)	1 (12.5%)	1 (33.33%)
Gross hematuria	4 (36.36%)	4 (50%)	0
Transient (resolved with conservative care)	3 (27.27%)	3 (37.5%)	0
Hematoma/clot evacuation	1 (9.09%)	1 (12.5%)	0
TUR cauterization	0	0	0
Postoperative stone/calcification requiring endoscopic removal	5 (45.45%)	2 (25%)	3 (100%)
Urethral stricture requiring dilation	1 (9.09%)	0	1 (33.33%)
Catheterization/retention	0	0	0

No formal statistical comparisons between radiation modalities were performed because of the small subgroup sizes. EBRT, external beam radiation therapy; AUS, artificial urinary sphincter; TUR, transurethral resection.

## Data Availability

The data presented in this study are available from the corresponding author upon reasonable request. The data are not publicly available due to patient privacy and ethical restrictions.

## Supplementary Materials

The following supporting information can be downloaded at: https://www.mdpi.com/article/10.3390/jcm15208168/s1, Table S1: Clinical, laboratory, and pathological characteristics; Table S2: Radiotherapy-related characteristics of the study cohort.
