# Supplementary material for: Channel-Focused Modified TURP for Post-Radiation Bladder Outlet Obstruction: A Case Series
Source: J Clin Med. 2026 Oct 20;15(20):8168. doi: 10.3390/jcm15208168 (PMC13706472; doi:10.3390/jcm15208168)
Supplement: Supplementary file 1 [file jcm-15-08168-s001.zip › jcm-4536225-supplementary.pdf]

Supplementary Table S1. Clinical, laboratory, and pathological characteristics.

Number	11
Age at RadTURP, years	75 (70.5 – 77)
Charlson comorbidity index, score	6 (5 – 6.5)
Prostate cancer and radiation therapy characteristics	
PSA at initial prostate cancer diagnosis, ng/mL	7.97 (5.69 – 8.76)
Gleason score	
≤7	10 (90.9%)
≥8	1 (9.1%)
NCCN risk category	
Low	6 (54.5%)
Intermediate	2 (18.2%)
High	2 (18.2%)
Very high	1 (9.1%)
Clinical T stage	
≤T2	10 (90.9%)
≥T3	1 (9.1%)
Androgen deprivation therapy (ADT) use, n (%)	10 (90.9%)
Duration of ADT, months	2 (2 – 7.5)

Data are number (%) and median (interquartile range).

RadTURP, modified transurethral resection of the prostate after radiation therapy; PSA, prostate-specific antigen; NCCN, National Comprehensive Cancer Network; ADT, androgen deprivation therapy.

Supplementary Table S2. Radiotherapy-related characteristics of the study cohort

Patient	RT modality	RT technique/type	Radiation dose/fractionation	Isotope	No. of needles	No. of seeds	RT–RadTURP interval
Brachy 1	Brachytherapy	Permanent seed implantation	NR	Iodine-125	24	104	21
Brachy 2	Brachytherapy	Permanent seed implantation	NR	Iodine-125	22	98	20
Brachy 3	Brachytherapy	Permanent seed implantation	NR	Iodine-125	20	87	12
Brachy 4	Brachytherapy	Permanent seed implantation	NR	Iodine-125	27	92	20
Brachy 5	Brachytherapy	Permanent seed implantation	NR	Iodine-125	18	70	13
Brachy 6	Brachytherapy	Permanent seed implantation	NR	Iodine-125	20	84	50
Brachy 7	Brachytherapy	Permanent seed implantation	NR	Iodine-125	22	100	42
Brachy 8	Brachytherapy	Permanent seed implantation	NR	Iodine-125	18	81	13
EBRT 1	EBRT	IMRT	60 Gy/20 fractions	–	–	–	13
EBRT 2	EBRT	External institution; details unavailable	Unavailable	–	–	–	6
EBRT 3	EBRT	External institution; details unavailable	Unavailable	–	–	–	4

RT details were extracted from available institutional and outside medical records. Detailed dose, fractionation, and treatment technique were unavailable for two patients who underwent EBRT at outside institutions. For brachytherapy patients, operative records documented iodine-125 permanent seed implantation and the numbers of implantation needles and seeds.

RT, radiation therapy; EBRT, external beam radiation therapy; IMRT, intensity-modulated radiation therapy; ADT, androgen deprivation therapy; RadTURP, modified transurethral resection of the prostate after radiation therapy; NR, not recorded.
